# Propagation of Upward and Downward Interface Acoustic Waves in Fused Silica/ZnO/SU-8/Fused Silica-Based Structures

**DOI:** 10.3390/s26010139

**Published:** 2025-12-25

**Authors:** Cinzia Caliendo, Massimiliano Benetti, Domenico Cannatà, Farouk Laidoudi

**Affiliations:** 1 Institute for Photonics and Nanotechnologies, IFN-CNR, Via del Fosso del Cavaliere 100, 00133 Rome, Italy; 2 Institute for Microelectronics and Microsystems, IMM-CNR, Via del Fosso del Cavaliere 100, 00133 Rome, Italy; massimiliano.benetti@cnr.it (M.B.); domenico.cannata@cnr.it (D.C.); 3 Research Center in Industrial Technologies CRTI, P.O. Box 64, Cheraga, Algiers 16014, Algeria; f.laidoudi19@gmail.com

**Keywords:** ZnO, fused silica, SAWs, downward IAW, upward IAW, IDTs, multifrequency delay line

## Abstract

**Highlights:**

**What are the main findings?**

- The main findings of this work is the coexistence of an upward- and downward-propagating interface acoustic wave (IAW) in SiO_2_/ZnO/IDT/SU-8/SiO_2_ which has been demonstrated both theoretically and experimentally. The former wave is predominantly localized in the SiO_2_/ZnO domain: it is excited within the SiO_2_/ZnO/IDT region and interacts with the SU-8/SiO_2_ overcoat. The latter is mainly confined to the SiO_2_/SU-8/ZnO domain: it is excited within the SiO_2_/SU-8/IDT/ZnO region and interacts with the SiO_2_ overcoat. The waves have quite different velocity, propagation loss and electroacoustic coupling coefficient.

**What is the implication of the main finding?**

- The findings presented herein establish a solid foundation for the development of next-generation, multi-frequency IAW-based devices, ranging from package-less acoustic components to microfluidic platforms and gas or optical sensors engineered for reliable operation in harsh environments.

**Abstract:**

The propagation of interfacial acoustic waves (IAWs) along a SiO_2_/ZnO/SU-8/SiO_2_ multilayer structure is theoretically predicted and experimentally validated. A two-dimensional finite-element analysis was performed using COMSOL Multiphysics, revealing that key IAW characteristics—such as the number of supported modes, propagation losses, and acoustic field distribution—are strongly influenced by the thickness of the intermediate SU-8 adhesive layer. In particular, the presence of the SU-8 layer enables the existence of IAW modes with opposite localization, namely upward- and downward-propagating IAWs. To validate the theoretical predictions, experimental measurements were carried out on delay lines fabricated on SiO_2_/ZnO/SU-8/SiO_2_ layered structures, revealing the propagation of three distinct IAW modes. The first two modes correspond to the downward and upward fundamental IAWs, while the third mode is a second-order mode identifiable as a downward leaky IAW (LIAW). The experimental results show excellent agreement with the theoretical predictions and establish a solid foundation for the future development of multifrequency IAW-based devices, including package-less acoustic components, microfluidic platforms, and gas and optical sensors designed for operation under harsh environmental conditions.

## 1. Introduction

Interface acoustic waves (IAWs) are a class of elastic waves whose propagation is confined to the planar boundary between two dissimilar elastic media, with amplitudes that decay exponentially into both solids [1]. When at least one of the two media is piezoelectric, the mechanical displacement field is intrinsically coupled to an accompanying electric field, enabling efficient electromechanical transduction. Owing to their low radiation of acoustic energy into the adjacent half-spaces—since the acoustic energy is concentrated at the interface and does not significantly leak into the surrounding bulk substrates—IAWs are particularly attractive for the realization of package-less acoustic devices. However, despite their potential, the identification of material combinations and structural configurations capable of sustaining low-loss, efficiently excitable IAWs with suitable electromechanical coupling remains a key challenge. In particular, it is still not fully clear which multilayer architectures best balance wave confinement, propagation loss, and practical fabrication constraints.

The early development of IAW-based devices was motivated by the need for acoustic components capable of operating reliably without hermetic sealing or protective packaging, thereby minimizing the impact of environmental interference on device performance [2,3,4]. Subsequently, single-crystal piezoelectric substrates were explored for IAW-based microfluidic applications, including continuous-flow submicron particle manipulation and the sorting of biological samples [5]. These approaches aimed, among other objectives, to mitigate acoustic energy leakage through polydimethylsiloxane (PDMS) layers, which represents a major limitation of conventional surface acoustic wave (SAW) microfluidic platforms [6,7,8]. The propagation of IAWs has been investigated in several material systems, including LiNbO_3_, PZT_4_, and LiTaO_3_, for various crystal cuts, propagation directions, and overcoat configurations [9,10,11,12]. By contrast, reference [13] reports a purely theoretical study in which IAW properties were calculated for different crystal orientations of quartz, lithium tantalate, and lithium niobate, considering IAW excitation at an interface located within a single material. Overall, while these studies highlight the advantages of IAWs—such as strong confinement and reduced radiation losses—they also reveal limitations related to material availability, fabrication complexity, and restricted flexibility in device design.

In the present work, we investigate a multilayer structure consisting of a fused silica substrate (500 µm thick) coated with a piezoelectric c-axis-oriented ZnO thin film (4 µm thick) and a fused silica overcoat (500 µm thick), the latter bonded through a thin SU-8 adhesive layer. SU-8 is a negative, epoxy-based photoresist widely used in micro- and nanofabrication for its mechanical stability, chemical resistance, and compatibility with thick-film processing [14]. While the fused silica/ZnO configuration is known to support higher-order SAWs—as previously demonstrated by the authors [15]—the introduction of a properly engineered SU-8/fused silica overcoat enables the excitation of multiple IAW modes. To the best of the authors’ knowledge, the propagation of IAWs in a structure consisting of a piezoelectric thin film sandwiched between two bulk fused silica substrates is reported here for the first time. The SU-8 layer plays a crucial role in enabling the coexistence of two fundamental IAW modes with opposite localization and different velocities, namely the upward- and downward-propagating IAWs, as well as a higher-order downward leaky IAW. A COMSOL Multiphysics 6.3-based numerical approach was developed to predict the existence and characteristics of these modes, and the experimental results show strong agreement with theoretical predictions. The demonstrated multifrequency operation, together with the package-less nature of the proposed structure, makes this platform particularly promising for acoustic components, microfluidic systems, and gas or optical sensors intended for operation in harsh environments.

## 2. Materials and Methods

A two-dimensional finite element analysis was carried out to simulate the IAW propagation in the SiO_2_/ZnO/SU-8/SiO_2_ structures, with ZnO 4 µm thick and wavelength λ = 80 µm, for different SU-8 layer thicknesses. The simulations revealed that key characteristics of IAW propagation—such as the number of modes, propagation losses, and field distribution—are strongly influenced by the thickness of the intermediate SU-8 adhesive layer.

**Figure 1:**
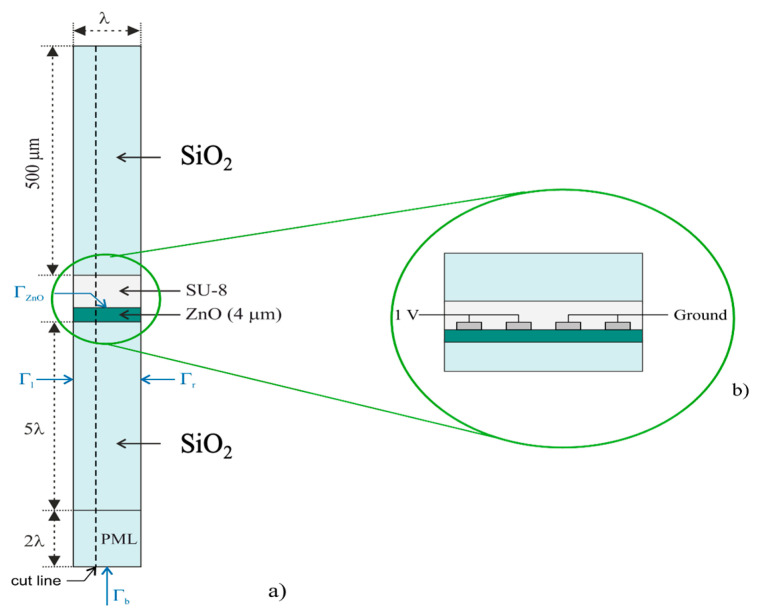
(**a**) The unit cell of SiO_2_/ZnO/SU-8/SiO_2_ structures used for eigenfrequency and frequency domain analysis; (**b**) a detailed view of IDTs.

Eigenfrequency and Frequency Domain studies were conducted using the commercial software COMSOL 6.3. The Solid Mechanics and Electrostatics interfaces were employed under the piezoelectric device Multiphysics coupling node to analyze the IAW propagation in the SiO_2_/ZnO/IDT/SU-8/SiO_2_ structure. FEM studies were conducted on a unit cell consisting of one-wavelength-wide (80 µm) domains of SiO_2_/ZnO/SU-8/SiO_2_. In particular, the models involve the following domains: the SiO_2_ substrate (5·λ thick), the ZnO piezoelectric material (4 µm thick), the SU-8 adhesion layer (from 1 to 20 µm thick), and the SiO_2_ overcoat (500 µm thick). A perfectly matched layer (PML) was used on the bottom of the substrate (2·λ thick) and periodic boundary conditions are applied to the lateral sides of the unit cell (Γ_l_ and Γ_r_), and the bottom boundary is fixed (Γ_b_). An electrically free or short boundary condition on the ZnO free surface (Γ_ZnO_) is used to calculate the electromechanical coupling coefficient see Figure 1a. For frequency domain studies, IDTs in spit finger configuration consisting in four Al electrodes (0.15 µm thick and λ/8 wide) were considered for the surface of the ZnO layer. Electrodes were connected to 1 V or grounded as shown in the detailed view reported in Figure 1b. Since the metal IDTs are much thinner than the other domains, numerical modelling of such a thin layer requires very small meshes which leads to high computational cost and time. To avoid this issue, the thin-layer option of COMSOL was used to represent the metal IDTs, thus allowing a substantial reduction in computational cost and time, without sacrificing the accuracy of the simulation.

**Figure 2:**
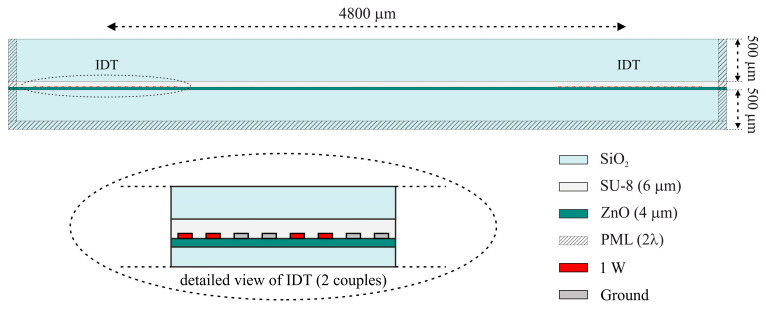
The SiO_2_/ZnO/IDT/SU-8/SiO_2_ configuration adopted to study the IAWs propagation and an enlarged view of the IDTs (red and gray colored).

The frequency domain study was also carried out to obtain the amplitude and phase of the scattering parameter S_21_ (the transmission coefficient) of the IAW-based delay lines. A 2D model was used for these simulations, consisting of a SiO_2_ substrate (500 µm thick), a ZnO film (4 µm thick) with two IDTs (launching and receiving) patterned on its surface, an SU-8 intermediate layer (6 µm thick), and a SiO_2_ overcoat (500 µm thick), as shown in Figure 2. PMLs were applied to the left, right, and bottom boundaries to suppress spurious reflections. The aluminum IDTs consisted of 80 fingers (λ/8 wide and 0.15 µm thick) arranged in a split-finger configuration (red and gray colored in Figure 2). The center-to-center distance between the input and output IDTs was 4800 µm. Two adjacent electrodes of both the input and output IDTs were excited with 1 W power, while all remaining fingers were grounded. The out-of-plane thickness of the computational domain was set equal to the finger overlap *w* used in the fabricated devices (*w* = 1568 µm).

In all simulations, the material constants of Al, SiO_2_, and ZnO were extracted from the materials library of COMSOL 6.3; the SU-8 constants (mass density 1190 kg/m^3^, Young modulus E = 4.02 GPa, Poisson ratio ν = 0.22, relative permittivity ε_r_ = 3) were extracted from reference [16]. The ZnO has 0.01 permittivity loss and 0.002 mechanical loss; SiO_2_ and SU-8 have isotropic mechanical loss equal to 0.01. An extremely fine mesh—consisting of physics-defined triangular elements automatically generated by COMSOL—was used for all FEM simulations.

### 2.1. Eigenfrequency Study’s Results

**Figure 3:**
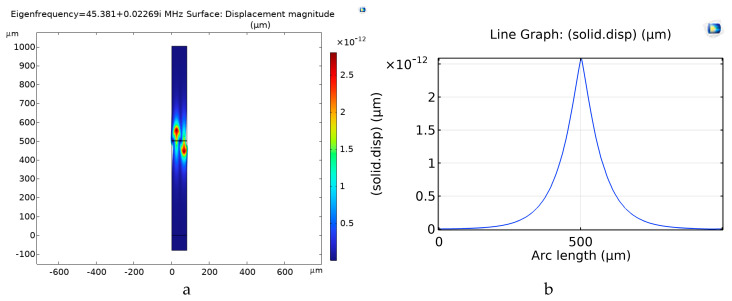
(**a**) The solid displacement and (**b**) the mechanical field profile of the IAW traveling in the SiO_2_/ZnO/SiO_2_ structure.

Stoneley waves propagate along the interface between two solid media, which may consist of a piezoelectric substrate with IDTs placed onto its surface and a non-piezoelectric overcoat, as in the case of the LiNbO_3_/IDT/Si structure, just to cite one example [10]. When the piezoelectric material is in thin-film form and is grown onto a non-piezoelectric substrate (such as sapphire, Si or SiO_2_, to cite just some examples), the resulting IAW-based structure consists of two bulk solid media (substrate and overcoat) separated by an intermediate layer, as in the SiO_2_/ZnO/IDT/SiO_2_ configuration. In this case the mechanical displacement associated with the IAW is confined to the mid-plane between the two bulk solids: the field is concentrated within the ZnO layer and symmetrically distributed in the two SiO_2_ substrates, as illustrated in Figure 3. The mechanical field profile of Figure 3b was estimated along a cut line starting from the lower side of the SiO_2_ substrate up to the upper side of the SiO_2_ overcoat.

**Figure 4:**
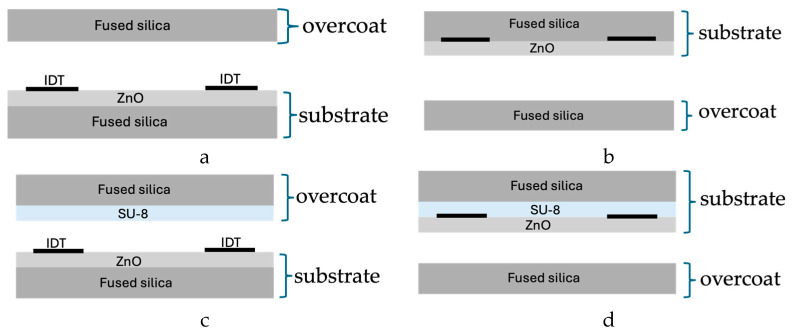
The schematic of (**a**) the downward and (**b**) upward IAW-based configurations for the SiO_2_/ZnO/IDT/SiO_2_ structure; the schematic of the (**c**) downward and (**d**) upward IAW-based configurations for the SiO_2_/ZnO/IDT/SU-8/SiO_2_ structure.

**Figure 5:**
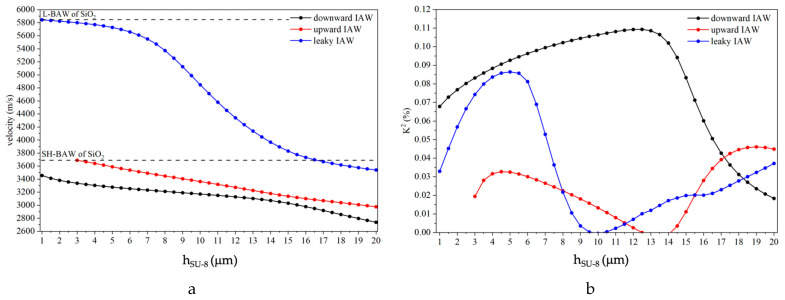
The phase velocity (**a**) and the K^2^ (**b**) vs h_SU-8_ curves of the downward (black dots) and upward (red dots) IAWs and of the LIAW (blue dots).

When an additional intermediate layer is introduced between the two substrates (as in the present SiO_2_/ZnO/IDT/SU-8/SiO_2_ structure), the conditions for the existence of one or more IAW modes must be re-evaluated, as will be discussed and illustrated in Figure 5. The presence of a thin SU-8 film gives rise to two distinct IAWs: one excited within the SiO_2_/ZnO/IDT region and interacting with the SU-8/SiO_2_ overcoat, and the other excited within the SiO_2_/SU-8/IDT/ZnO region and interacting with the SiO_2_ overcoat. The former is predominantly localized in the SiO_2_/ZnO domain (hereafter referred to as the downward IAW), while the latter is mainly confined to the SiO_2_/SU-8/ZnO domain (hereafter referred to as the upward IAW). Figure 4a–d show the schematic of the downward and upward IAW-based configurations for the SiO_2_/ZnO/IDT/SiO_2_ and SiO_2_/ZnO/IDT/SU-8/SiO_2_ structures.

Moreover, since the basic SAW structure—namely the SiO_2_/ZnO/IDT/AIR configuration—supports the propagation of higher-order surface waves [15], including Rayleigh, Sezawa, and others, the SiO_2_/ZnO/IDT/SU-8/SiO_2_ structure investigated here also supports the propagation of higher-order IAWs. Each of these modes should, in principle, exhibit an upward counterpart, unless the latter becomes too lossy to be effectively excited or detected.

Of course, downward and upward IAWs also exist in the SiO_2_/ZnO/IDT/SiO_2_ structure: these two modes correspond to two different electroacoustic coupling configurations—namely, the SiO_2_/ZnO/IDT configuration and the SiO_2_/IDT/ZnO configuration—both covered by the same SiO_2_ overcoat. However, since these two SAW coupling configurations exhibit markedly different phase velocities and electroacoustic coupling coefficients K^2^ (0.16% for SiO_2_/ZnO/IDT and 0.0003% for SiO_2_/IDT/ZnO), the upward IAW is only weakly excited and thus barely detectable compared with the downward IAW. By contrast, when an SU-8 intermediate layer of the proper thickness h_SU-8_ is introduced between the two substrates, the two IAWs acquire distinct velocity and sufficiently large K^2^ to be clearly separated and experimentally observed.

The phase velocities of the IAW modes were calculated for different SU-8 layer thicknesses (from 1 up to 20 um) at a fixed wavelength (λ = 80 µm) by performing a parameter sweep in the eigenfrequency study. The mode velocity was determined as *v* = *λ* × *f*, where λ = 80 µm denotes the wavelength and *f* the corresponding eigenfrequency. Figure 5a shows the phase velocity dispersion curve of the upward and downward IAWs, as well as that of the LIAW. From Figure 5a it can be noticed that the upward IAW and the LIAW have different cut off velocities corresponding to the shear and longitudinal BAW velocity in the SiO_2_ material.

The phase velocity of the three IAWs decreases with increasing h_SU-8_. While the downward IAW exists for all the studied SU-8 thicknesses (black dots), the velocity of the upward IAW (red dots) has a cut off at h_SU-8_ = 3 μm which corresponds to the shear horizontal bulk acoustic wave (SHBAW) in the SiO_2_ (3687.5 m/s). For h_SU-8_ < 3 μm the upward IAW leaks energy into the overcoat and becomes hardly observable; as h_SU-8_ increases, the acoustic energy becomes increasingly confined at the interface and the wave becomes measurable. The upward wave is faster than the downward one since its upper velocity limit—the SHBAW velocity of the bare SiO_2_ overcoat material—is higher than that of the SU-8/SiO_2_. The upper velocity limit of the LIAW is the longitudinal BAW of the SiO_2_ (5848.1 m/s): the downward IAW exists for all the studied h_su-8_ values (blue dots) but it becomes extremely lossy for h_SU-8_ > 8 μm, while the upward LIAW is highly leaky and not distinguishable.

The electromechanical coupling coefficient (K^2^) quantifies the efficiency of the electrical-to-acoustic energy conversion; it was extracted from the eigenfrequency results by computing the phase velocities of the modes under electrically free (*v_free_*) and short-circuited (*v_short_*) boundary conditions on the ZnO free surface, using the approximate relation: *K*^2^ = 2·(*v_free_* − *v_short_*) / *v_free_*. Figure 5b shows the K^2^ dispersion curves for the upward and downward IAWs and of the LIAW. The K^2^ of the downward mode remains nearly constant as h_SU-8_ increases, but it begins to decrease for thicknesses exceeding approximately 12 µm. In contrast, the K^2^ of the upward IAW is almost negligible at very small adhesive-layer thicknesses, as the mode is weak and close to its cutoff condition. With increasing h_SU-8_, K^2^ progressively increases and attains a maximum value; thereafter, it decreases to nearly zero and subsequently rises again, eventually exceeding the K^2^ of the downward mode. The K^2^ of the LIAW increases for increasing h_SU-8_ up to 5 μm and then it decreases down to zero; further increasing h_SU-8_ corresponds to the wave propagation mostly trapped inside the adhesive layer.

### 2.2. Frequency Domain Study’s Results

**Figure 6:**
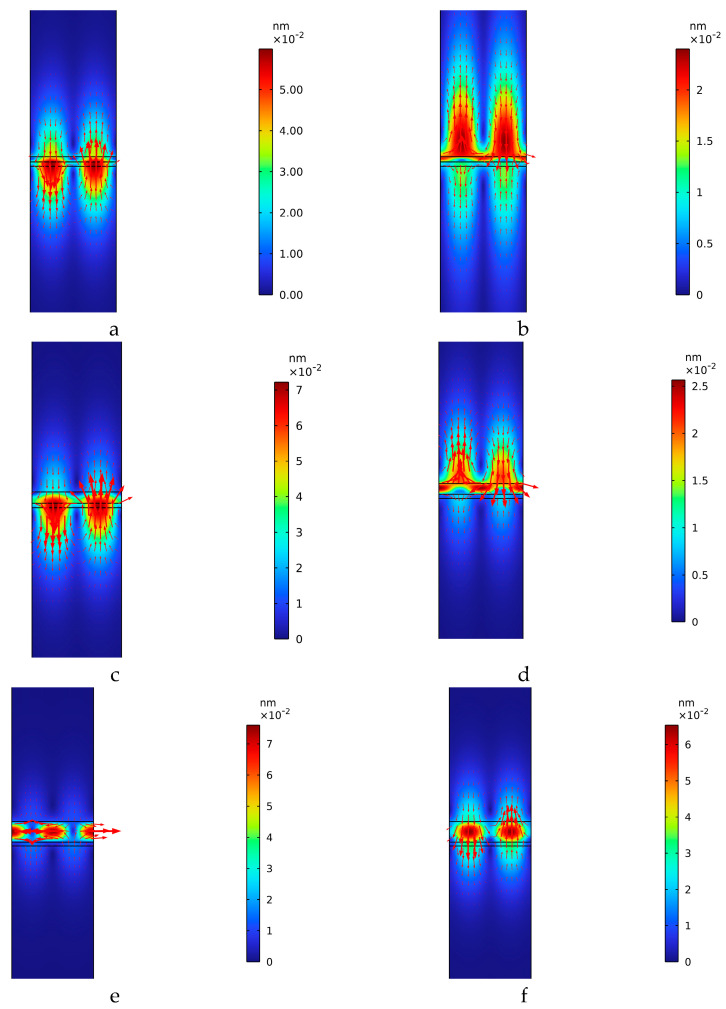
The solid displacement of the downward (**a**,**c**,**e**) and upward (**b**,**d**,**f**), for different SU-8 layer thicknesses (5, 10, and 20 μm).

The frequency domain study was performed to calculate the mechanical displacement field of the two IAWs for different SU-8 layer thicknesses, as shown in Figure 6 where the red arrows point in the direction of the local displacement vector; the arrow length is proportional to the displacement amplitude. With increasing h_SU-8_ (for example, for a 20 µm thick SU-8 layer), the mechanical displacement of the downward and upward IAWs evolves into a longitudinal mode and a shear-vertical mode, respectively, with the former being strongly confined within the adhesive layer.

**Figure 7:**
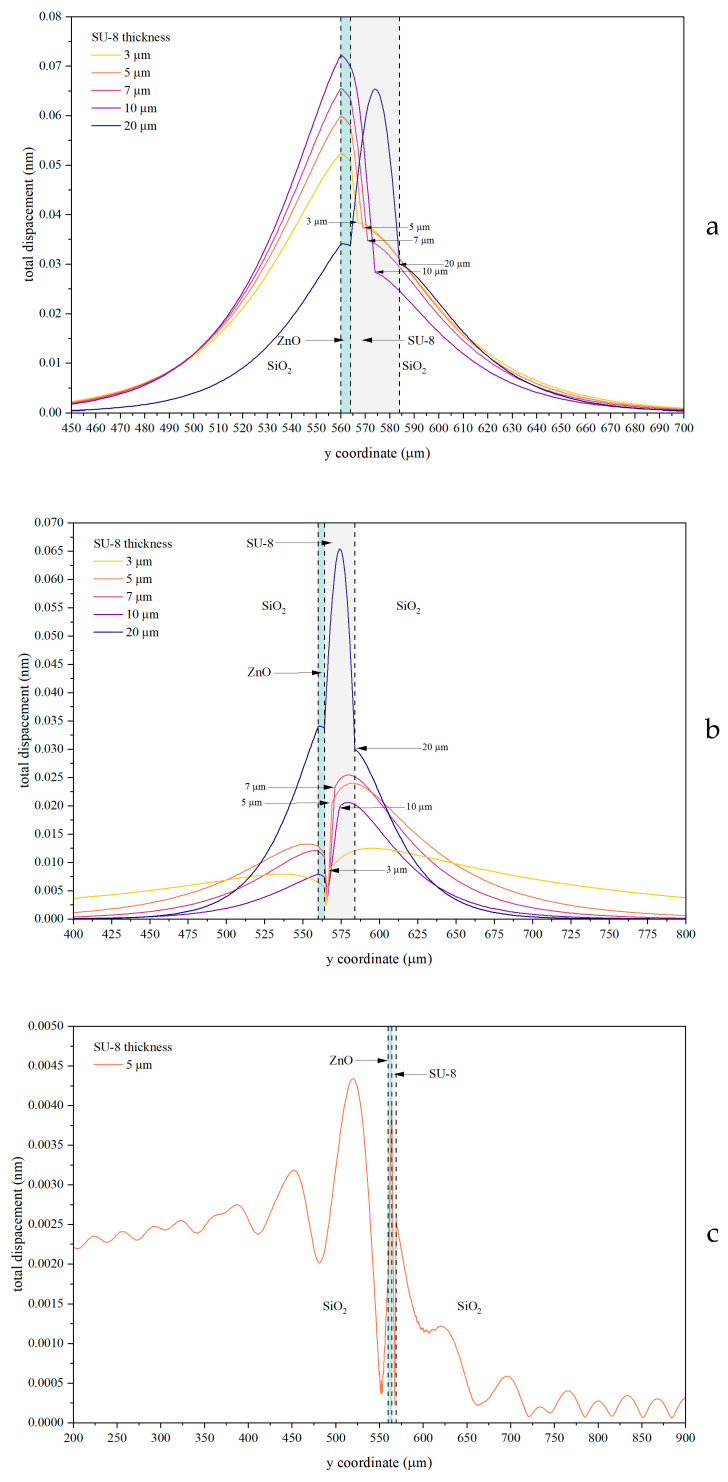
The solid displacement profile of the downward (**a**) and upward (**b**) IAWs, for different SU-8 layer thicknesses, and of the LIAW (**c**) for h_SU-8_ = 5 μm.

These features are even more evident in Figure 7, which reports the spatial profiles of the mechanical displacement along a cut line (indicated in Figure 1a) for several SU-8 layer thicknesses.

As shown in Figure 7, the field amplitude exhibits a strong dependence on the SU-8 layer thickness, with each of the three waves displaying a distinct trend. The downward IAW is mainly localized within the SiO_2_/ZnO/SU-8 region and exhibits a small evanescent tail extending into the SiO_2_ overcoat, whereas the upward IAW is predominantly confined within the ZnO/SU-8/SiO_2_ region with a weak tail penetrating into the SiO_2_ substrate. As the SU-8 thickness increases from 3 to 10 µm, the acoustic field amplitude increases and the mode confinement at the corresponding interfaces becomes stronger. By contrast, at an SU-8 thickness of 20 µm the acoustic field becomes predominantly confined within the adhesive layer and decays exponentially into both substrates evidencing evanescent penetration away from the guiding polymer layer. Figure 7c shows the displacement profile of the LIAW for an SU-8 thickness of 5 µm. The acoustic field exhibits a pronounced localization at the ZnO/SU-8 interface, while decaying away from it into both SiO_2_ substrates. The oscillatory behavior observed, especially within the substrate, indicates a small but non-negligible leakage of acoustic energy away from the guiding interface, consistent with partial radiation into the bulk of the substrate.

**Figure 8:**
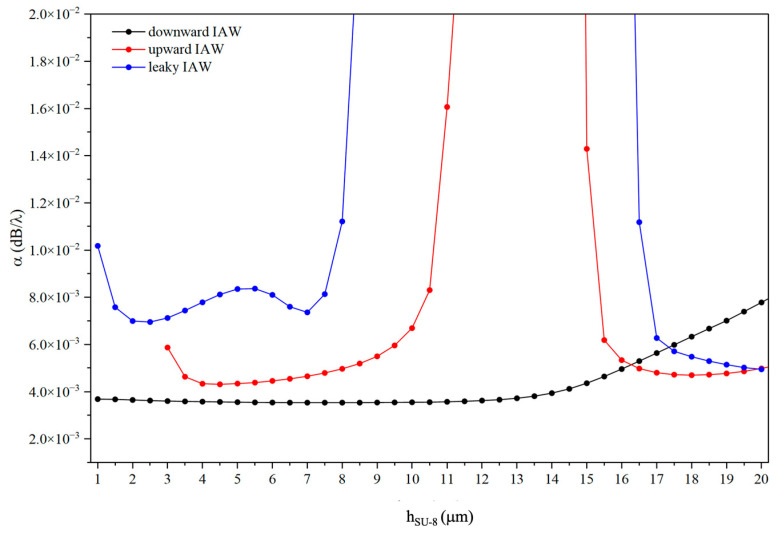
The propagation loss of the downward and upward IAWs and of the LIAW vs h_SU-8_.

The admittance Y vs frequency curves of the unit cell were calculated for different SU-8 layer thicknesses by applying 1 V to the electrodes; then the propagation loss of the waves was calculated using the following relation:

(1) $$\alpha =\frac{\omega }{2Qv_{g}},$$

where ω=2πf, $v_{g}=v_{ph}+\frac{h}{\lambda }\frac{\partial v_{ph}}{\partial \left(h/\lambda \right)}$ is the group velocity, $v_{ph}$ is the phase velocity, and $Q=\frac{f_{r}}{\Delta f_{3dB}}$ [17]. Here, $f_{r}$ and $\Delta f_{3dB}$ were extracted from the real part of the admittance (*Y*) vs. frequency curves obtained through a frequency domain study: $f_{r}$ and $\Delta f_{3dB}$ correspond to the resonance peak and the half-power bandwidth, respectively. Figure 8 shows the calculated propagation loss α (dB/λ) as a function of the SU-8 thickness for the downward IAW, upward IAW, and leaky IAW modes.

The downward IAW exhibits relatively low and weakly thickness-dependent losses over a wide SU-8 thickness range, consistent with the strong and stable confinement at the SiO_2_/ZnO/SU-8 interface observed in Figure 7a. In contrast, the upward IAW shows a pronounced dependence on the SU-8 thickness, with sharp loss peaks that correlate with the redistribution of the acoustic field and the increased penetration into the substrates, as evidenced by the field profiles in Figure 7b. The leaky IAW systematically exhibits higher attenuation and abrupt loss variations, in agreement with the oscillatory field behavior and partial radiation into the bulk shown in Figure 7c. Overall, these results demonstrate that the propagation losses are directly governed by the SU-8-induced modification of the acoustic field confinement and leakage mechanisms.

**Figure 9:**
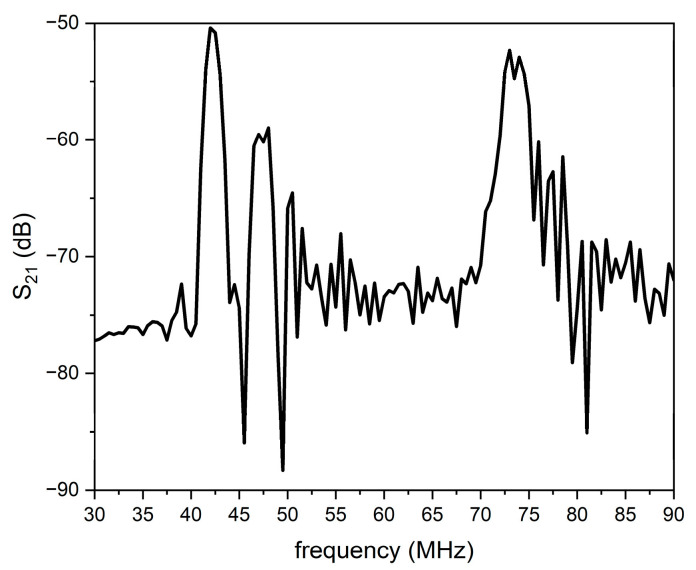
The S_21_ vs. frequency curve of the IAW-based delay line for 6 μm thick SU-8 layer.

Figure 9 shows the scattering parameter S_21_ vs. frequency curve of the prototype delay line reported in Figure 2, for a 6 µm thick SU-8 layer.

IAWs are excited at approximately 40, 45, and 72 MHz; the first two modes are the downward and upward IAWs, the third mode is a leaky IAW (LIAW), which radiates energy into the bulk of the substrate and the overcoat. The third mode is faster than the other modes as its velocity approaches the velocity of the longitudinal BAW in the SiO_2_; although the LIAW leaks energy into the SiO_2_ substrates, it still preserves enough energy to be reliably detected by the receiving IDT.

The thickness of the SU-8 layer was studied in a wide range (from a few up to tens of micrometers) but finally a 6 µm thickness was chosen as it ensures that the acoustic energy remains primarily confined within the ZnO layer and the insertion losses are not too big.

It should be emphasized that the theoretical calculations are subject to uncertainties arising from both the SU-8 material constants and the intrinsic losses of the materials composing the stack. Notably, the material properties of SU-8 reported in the literature show considerable variability, leading to non-negligible discrepancies among different studies.

## 3. Experimental

### 3.1. Delay Line Fabrication

**Figure 10:**
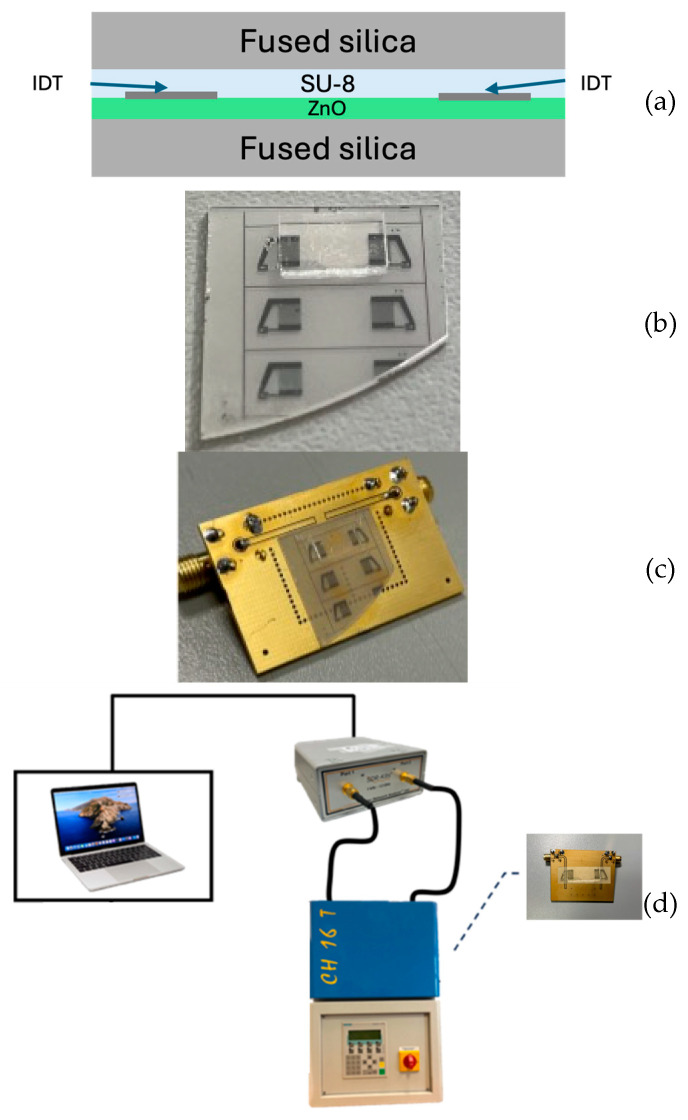
(**a**) A schematic of the stacked structure; (**b**) The photo of the just fabricated IAW delay line; (**c**) A photo of the device fixed to the PCB; (**d**) A schematic view of the experimental setup.

Piezoelectric c-axis-oriented ZnO films, approximately 4 µm thick, were grown on fused silica substrates using RF reactive magnetron sputtering. A delay line consisting of interdigital transducers (IDTs) with an 80 µm periodicity in a split-finger configuration was then fabricated by a lift-off process, following the technological procedure previously described by the authors in [15]. The resulting SiO_2_/ZnO/IDT structure was fixed to the PCB using an epoxy adhesive and electrically connected through aluminum wire bonding. The SU-8 3005 layer was spin-coated onto the fused silica overcoat following the procedure reported in Refs. [10,11]. The SU-8-coated substrate was then brought into contact with the SiO_2_/ZnO/IDT substrate and subjected to mild pressure and a soft bake at 95 °C to activate SU-8 adhesion and promote solvent evaporation, resulting in a stable adhesive-bonded structure. Since the SU-8 layer was compressed during bonding, its actual thickness is not precisely known; however, based on the manufacturer’s specifications for spin-coating speed versus thickness, and considering the mild pressure applied—which may reduce the film thickness by a few percent—we estimate its final thickness to be approximately 5–6 µm. Figure 10a–d show a schematic of the stacked structure, a photo of the just fabricated IAW-based delay line and of the device fixed to the PCB, and the experimental setup used to test the devices.

### 3.2. IAW-Based Device Test

**Figure 11:**
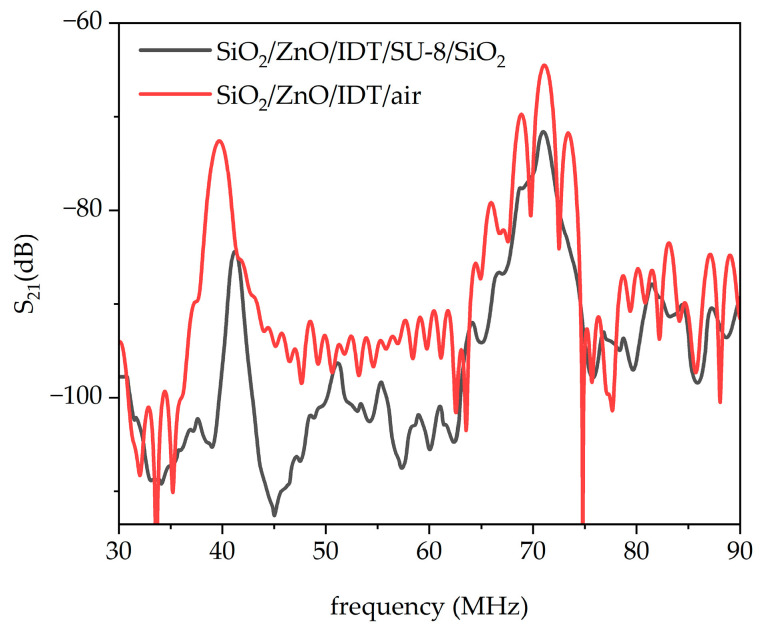
The S_21_ vs. frequency curves for the SAW and IAW-based delay line. For all the figures, the ZnO is 4 μm thick and λ is 80 μm.

The scattering parameter S_21_ of the untuned IAW delay lines was measured in the frequency domain by using a vector network analyzer (VNA, DG8SAQ VNWA 3 from SDR-Kits, Melksham, UK) which was connected to the PC for real-time acquisition; measures were performed in the dark with controlled humidity, as shown in Figure 10c. Figure 11 reports the untuned, uncalibrated frequency domain $S_{21}$ response of the SiO_2_/ZnO/IDT/SU-8/SiO_2_ structure, compared with the reference SiO_2_/ZnO/IDT/air SAW delay line.

**Figure 12:**
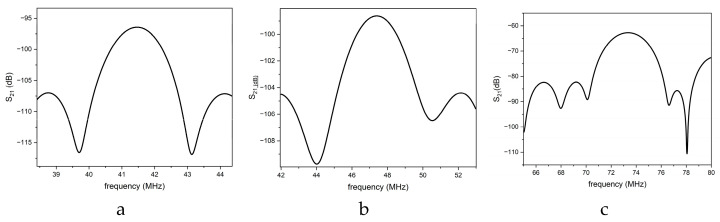
The S_12_ vs. frequency curves of the (**a**) downward IAW, (**b**) upward IAW, and (**c**) LIAW.

In Figure 11 the downward and leaky IAWs (black curve) appear at frequencies higher than that of the Rayleigh (at about 39 MHz) and leaky Sezawa (at about 70 MHz) modes (red curve), confirming their higher phase velocities. Moreover, the presence of the upward-propagating IAW is clearly evidenced. The S_21_ curves reported in Figure 12a–c were obtained by applying a time-gating procedure to suppress spurious contributions in the time domain response. The gate center was positioned at the maximum of the time response, while the gate span was carefully adjusted to include the full set of sidelobes associated with the acoustic signal of interest. The gated time domain data were subsequently transformed back into the frequency domain through an inverse Fourier transform, yielding an idealized frequency response with reduced parasitic interference. Measurements were performed with the VNA calibrated using a two-port short–open–load–through (SOLT) calibration up to the coaxial cables. This calibration excludes the test fixture, which consists of two SMA connectors mounted on the PCB on which the device was fixed.

The downward IAW is observed at approximately 42 MHz, the upward IAW at about 47 MHz, and the LIAW at around 73 MHz. These frequencies are higher than those of the Rayleigh and leaky Sezawa modes propagating in the untuned SiO_2_/ZnO/IDT delay line (about 40 and 71 MHz, with insertion losses of roughly −60 dB), since the IAWs propagate faster than the corresponding SAWs. The measured IAW frequencies are consistent with an SU-8 layer thickness of approximately 6 µm, in agreement with the data reported in Figure 5. The propagation loss of the IAWs estimated from the peak value of the $S_{21}\left(f\right)$ curves is larger than the theoretically calculated losses of Figure 9. This discrepancy may arise from (i) technological factors—such as non-ideal ZnO film quality (e.g., crystallographic texture, residual stress, thickness non-uniformity), imperfect Al IDT fabrication (e.g., edge roughness, resist residues, contact resistance), and process-induced defects at the interfaces—or (ii) modeling-related limitations, including uncertainties in the SU-8 material constants (especially viscoelastic parameters), incomplete inclusion of attenuation mechanisms (bulk damping, thermoelastic losses, electrical and dielectric losses), and non-ideal boundary conditions or interface conditions (e.g., imperfect bonding, finite stiffness/roughness at the SU-8 interfaces).

As further confirmation of the interface-guided nature of the waves and of the field confinement discussed above, the IAW propagation was experimentally tested under liquid loading conditions. When a few drops of deionized water were placed on the free surface of the overcoat during device operation, no measurable perturbation of the IAW response was observed, in clear contrast to conventional SAWs, which are strongly affected by surface loading. This behavior is consistent with the simulated displacement profiles, which show an exponential decay of the acoustic field away from the guiding interface and negligible displacement at the free surface. Altogether, these observations confirm that the IAW energy is not radiated toward the free surface, in full agreement with numerical simulations.

## 4. Discussion

Interfacial waves occur at the boundary of two infinite dissimilar materials and, unlike the SAWs, exist only under special circumstances. An important limitation of SAW-based devices is that most of the acoustic energy is confined near the substrate surface. As a consequence, their operating frequency is highly sensitive to surface conditions—such as ambient temperature or pressure variations, dust deposition, wetness, contamination or any other surface-related effects—which degrade the frequency stability of the SAW device (resonator, filter or delay line). For this reason, the surface of a SAW device must typically be covered or passivated to protect the wave from contamination, a requirement that often leads to costly packaging and sealing solutions. This limitation can be overcome by employing IAW-based devices, which are intrinsically insensitive to surface perturbations while still being excited and detected through standard interdigital transducers, as in conventional SAW devices. Since the Stoneley wave, derived from SAW technology, is also supported by planar structures, it can be fabricated using standard photolithographic processes. Consequently, Stoneley wave devices can be produced at manufacturing costs comparable to those of integrated circuits. This is particularly significant, as it opens the way to a new class of acoustic resonators and sensors with stability superior to traditional SAW devices. Unlike layered SAW structures, Stoneley wave devices are naturally well passivated and do not require hermetic sealing, resulting in improved long-term stability and reduced fabrication cost.

In the available literature, most reported IAW-based devices rely on single-crystal piezoelectric substrates—such as LiNbO_3_ and LiTaO_3_—coupled with bulk overcoats, whereas only a limited number of studies address configurations based on piezoelectric thin films. In the case of the SiO_2_/ZnO/IDT/SU-8/SiO_2_ configuration, the most distinctive feature is the possibility of multifrequency operation, enabled by the coexistence of upward and downward IAWs localized at different interfaces. This allows the simultaneous excitation of multiple acoustic modes and strong acoustic confinement in selected regions of the structure, making this architecture particularly attractive for sensing applications and for package-less devices. By contrast, IAW devices based on bulk piezoelectric substrates typically exhibit a more robust response, generally dominated by a single IAW mode; multifrequency operation in these systems has been demonstrated through the excitation of upper and lower IAWs, as reported in the literature [10,11]. The use of single-crystal piezoelectric materials ensures well-established and reliable material parameters, resulting in more predictable electroacoustic behavior. The main drawback of bulk-based configurations is their high material cost, which limits scalability and large-area or low-cost implementations. Overall, the comparison indicates that thin-film IAW structures offer greater functional flexibility and integration potential, at the expense of increased sensitivity to technological parameters, whereas bulk piezoelectric solutions prioritize maturity and reproducibility, but with higher costs and reduced versatility. Table 1 summarizes the key parameters of these two approaches and outlines their respective advantages and limitations, highlighting the trade-offs between bulk and thin-film piezoelectric IAW implementations.

In reference [18], both theoretical and experimental studies are reported on the propagation of IAWs in SiO_2_/ZnO/SiO_2_ structures, consisting of a thin ZnO piezoelectric film and employing 10 µm wavelength IDTs embedded beneath a 3.8 µm thick ZnO layer. No adhesive layer was used, as the 23 µm thick SiO_2_ overcoat was directly deposited onto the ZnO surface. The configuration investigated in the present work, although also based on a piezoelectric ZnO film, differs from the previous design in several essential aspects. First, the IDTs are patterned on top of the ZnO layer rather than embedded beneath it. Second, the overcoat consists of a bulk SiO_2_ substrate (500 µm thick) instead of a deposited SiO_2_ thick layer. Third, an SU-8 adhesive film is inserted between the SiO_2_ overcoat and the SiO_2_/ZnO/IDT stack. This approach enables a simpler, faster, and lower-cost fabrication process, although at the expense of additional acoustic losses introduced by the SU-8 layer above the IDTs. Moreover, placing the IDTs on top of the ZnO film prevents potential modifications of the ZnO microstructure—such as changes in crystal orientation, grain size, density, or surface roughness—that may occur when the electrodes are buried beneath the piezoelectric layer.

The obtained results indicate that the clamped boundary condition of the standard SAW-based SiO_2_/ZnO/IDT structure—which supports the propagation of higher-order SAW modes—effectively shifts the resonance peaks toward higher frequencies. Importantly, the introduction of the SU-8 adhesive layer gives rise to the propagation of two separate interface acoustic modes—the downward and upward IAWs—each characterized by different localization properties, phase velocity, and electromechanical coefficient. The downward mode is predominantly localized at the SiO_2_/ZnO interface and the upward mode is mainly confined at the SiO_2_/SU-8/ZnO interface. The former mode makes use of the SU-8/SiO_2_ overcoat, while the latter employs the bare fused silica overcoat and exhibits a cutoff velocity approximately equal to the transverse BAW velocity of the SiO_2_. In addition to the down- and upward IAWs, also a downward low-loss LIAW also propagates at velocity close to that of the longitudinal BAW of the SiO_2_, while the upward LIAW is too lossy to be detected for the SU-8 thickness range tested.

The presence of the SU-8 layer is essential to ensure the bonding between the substrate and the overcoat; however, it also represents a structural weakness that critically affects wave propagation, as also observed also in reference [19] where the authors employed an α-cyanoacrylate instant adhesive to study boundary-wave propagation along PZT/adhesive/PZT and PZT/adhesive/Corning-glass structures. In that work, the discrepancy between theoretical predictions and experimental results was attributed to the non-uniformity of the adhesive layer. In reference [20] a low-temperature hetero-bonding process enabling the attachment of lithium niobate onto silicon is demonstrated using a thin (less than 1 µm) spin-coated SU-8 layer. Although wave excitation and propagation at the LiNbO_3_/Si interface were successfully shown, this material combination and bonding technique are not suitable for practical applications due to the high losses introduced by the adhesive. Ongoing research is therefore focused on reducing these losses by employing stiffer bonding layers, such as spin-on-glass (SoG).

In our case, although SU-8 provides good process compatibility and ease of use, its mechanical losses and the uncertainty of its effective thickness after bonding introduce non-idealities that are difficult to fully capture in the simulations. In the present case study, SU-8 layers with increased thickness correspond to lower wave velocities, which is consistent with a mass-loading effect. Unfortunately, the IAW propagation loss measured is higher than the theoretical prediction but the discrepancy between theoretical and experimental results may arise from several factors:

1. the SU-8 material constants used in the simulations are affected by some uncertainty;
2. the SU-8 layer is modeled as an elastic solid rather than a viscoelastic material;
3. the SU-8 layer buried beneath the SiO_2_ overcoat may be thinner than the freshly spin-coated film, because the SiO_2_/SU-8 overcoat is pressed against the SiO_2_/ZnO substrate to ensure the absence of trapped air.

Looking ahead, several strategies may further enhance device performance: (i) exploring alternative adhesive materials with improved mechanical stiffness and reduced viscoelastic losses; (ii) optimizing the IDT metal type and thickness to increase the electroacoustic coupling coefficient K^2^; and (iii) implementing alternative electroacoustic coupling configurations, such as IDTs operating against a floating metal plane placed on the opposite side of the ZnO layer. Combining these approaches may enable the realization of high-performance IAW-based devices with enhanced confinement, higher resonant frequencies, and reduced attenuation.

## 5. Conclusions

Interface acoustic waves (IAWs) were excited and detected by interdigital transducers and propagated along a ZnO layer sandwiched between two bulk fused silica substrates. Two distinct types of IAWs were experimentally observed and theoretically predicted, referred to as downward and upward IAWs. The downward IAW originates from electroacoustic excitation in the SiO_2_/ZnO medium and interacts with the SU-8/SiO_2_ overcoat, whereas the upward IAW is generated by excitation in the SiO_2_/SU-8/ZnO medium and interacts with the bare fused silica overcoat. As a consequence—and as experimentally demonstrated—the upward IAW propagating in the SiO_2_/ZnO/SU-8/SiO_2_ structure exhibits a higher phase velocity than the downward IAW, since its upper velocity limit, determined by the shear-horizontal (SH) wave velocity of bare SiO_2_, exceeds that of the SU-8/SiO_2_ medium.

For completeness, it is noted that the leaky interface acoustic wave (LIAW) originates from the electroacoustic excitation of the leaky Sezawa mode propagating in the SiO_2_/ZnO medium and interacting with the SU-8/SiO_2_ overcoat. In principle, both upward and downward LIAWs may propagate in the multilayer structure; however, their excitation efficiencies differ significantly, such that only the downward LIAW was experimentally observed.

Because the mechanical displacement and accompanying electric fields of the IAWs are predominantly confined within the ZnO layer, the upper and lower SiO_2_ substrates act as effective protective layers. Owing to the wide bandgap of ZnO (about 3.6 eV), these multilayer architectures are therefore well suited not only for package-less acoustic devices but also for ultraviolet (UV) light sensing applications intended for operation in harsh environments.

Despite these advantages, several challenges remain for IAW-based devices, particularly in thin-film configurations. Device performance is strongly dependent on the crystalline quality and thickness uniformity of the piezoelectric ZnO layer, as well as on the poorly controlled viscoelastic properties of polymeric adhesive layers such as SU-8. Loss mechanisms—including viscoelastic damping in polymers, interface imperfections, and electrical and mechanical losses—are difficult to quantify and often dominate overall attenuation. In addition, fabrication reproducibility remains a critical issue, as small variations in layer thicknesses, interface quality, or IDT geometry can lead to significant device-to-device variability in frequency response and insertion loss. From a modeling perspective, theoretical and numerical approaches typically rely on idealized material parameters and boundary conditions, frequently resulting in an underestimation of propagation losses and discrepancies with experimental results. Moreover, the coexistence of multiple IAW modes (upward, downward, and leaky) complicates mode identification, selective excitation, and data interpretation. Finally, long-term stability and reliability may be affected by polymer aging, temperature dependence, and environmental exposure.

Overall, the central challenge lies in achieving an optimal balance between strong acoustic confinement and multifunctionality on one hand and low loss, reproducibility, and reliable modeling on the other, while preserving the intrinsic integration advantages of thin-film IAW platforms.

Based on the results of this first-phase study, future research can be directed along several complementary lines aimed at transforming thin-film IAW devices from proof-of-concept demonstrations into robust, low-loss, and highly integrated platforms suitable for multifunctional sensing and advanced acoustic signal processing. Key research directions include: (i) the development of advanced adhesive and intermediate layers, through the replacement of SU-8 with alternative materials exhibiting higher mechanical stiffness and lower viscoelastic losses or through hybrid adhesive solutions; (ii) improved material characterization, involving the systematic extraction of elastic, piezoelectric, dielectric, and loss parameters of thin films and polymers as a function of frequency and temperature, enabling more predictive modeling; (iii) optimized electroacoustic coupling schemes, achieved by refining IDT designs (metal type, thickness, apodization) and exploring alternative electrical boundary conditions, such as floating or grounded metal planes, to enhance the electroacoustic coupling coefficient $K^{2}$ and reduce insertion losses; (iv) improved mode selectivity and control, favoring the excitation of either upward or downward IAWs while suppressing leaky modes; (v) enhanced integration and scalability through monolithic integration with microfluidic, photonic, or electronic components; and (vi) systematic investigations of reliability and environmental robustness under harsh operating conditions.

The improved understanding and optimization of IAW propagation in SiO_2_/ZnO/SU-8/SiO_2_ multilayer structures also enable several promising application scenarios. The strong confinement of acoustic energy at well-defined interfaces, combined with the possibility of tailoring wave velocity and attenuation via the adhesive layer and the overcoat material, makes these devices particularly attractive for sensing applications. Potential applications include UV-light sensing, gas and vapor detection exploiting ZnO’s selective adsorption properties, and integrated lab-on-chip systems enabling compact, multiparameter sensing. Ongoing work is focused on further improving device performance by investigating alternative adhesive materials and adopting advanced electrical boundary conditions.

## Figures and Tables

**Table 1:** The key parameters and the advantages and limitations of the IAW configurations.

IAW Configuration	Key Parameters	Advantages	Limitations
SiO_2_/ZnO/IDT/SU-8/SiO_2_	Multifrequency operation; coexistence of upward and downward IAWs; thin piezoelectric film technology.	Simultaneous excitation of multiple IAW modes; strong acoustic confinement at different interfaces; package-less architecture; suitability for sensing applications.	Performance affected by SU-8 thickness uncertainty and viscoelastic losses.
piezoelectric substrate/SU-8/overcoat (e.g., LiNbO_3_/overcoat)	Single dominant IAW mode; fixed confinement interface; bulk piezoelectric single crystal.	Well-established material parameters; lower fabrication complexity; multifrequency operation based on upper and lower IAWs [10,11].	High cots.

## Data Availability

The original contributions presented in this study are included in the article. Further inquiries can be directed to the corresponding author.
